# A multicentric survey and single-centre observational study of usage behaviour of sinks in intensive care: training is needed to minimize risk

**DOI:** 10.1186/s13756-024-01493-3

**Published:** 2024-11-17

**Authors:** Giovanni-Battista Fucini, Robert Abe, Elke Lemke, Petra Gastmeier

**Affiliations:** Institute of Hygiene and Environmental Medicine, Charité – Universitätsmedizin Berlin, corporate member of Freie Universität Berlin and Humboldt-Universität zu Berlin, Hindenburgdamm 27, 12203 Berlin, Germany

**Keywords:** Sinks, Infection prevention, ICU, Hand hygiene

## Abstract

**Introduction:**

Sinks have been introduced near patients to improve hand hygiene as part of infection prevention and control measures. However, sinks are a known reservoir for gram-negative bacterial pathogens in particular and their removal to prevent bacterial infections in intensive care patients is currently recommended by several international guidelines.

**Methods:**

Healthcare workers (HCWs) in 15 intensive care units (ICUs) in Germany were given the opportunity to complete an anonymous survey on the use of sinks between August 2022 and January 2023. Observations were then made in three participating ICUs to determine the frequency and reason for contact with the sink.

**Results:**

258 questionnaires were returned (nurses 87%). 90% found it useful to very useful to have a sink in the patient room, and 56% reported using it daily for hand hygiene. We observed 33 contacts between nurses and sinks over 17 h. In 20/33 (60%) cases, the sink was used for waste disposal. In 3/33 (10%) it was used for hand washing.

**Discussion:**

Sinks are still used for daily care in intensive care units. Educational Interventions in existing buildings to minimise risk through “sink hygiene” (i.e. separation of sinks for water disposal and uptake) can make an important contribution to infection prevention.

## Introduction

Plumbing systems always contain a biofilm, a heterogeneous mixture of bacterial communities surrounded by a protective matrix. Biofilms can form especially in areas with slow flow rates, warm temperatures and low residual disinfection. Due to the size and complexity of water distribution systems in hospitals, this is often the case at the most remote points of the system, e.g. sinks and showers [1, 2]. Therefore, sinks and drains in hospitals can become reservoirs for bacterial pathogens [1].

Prolonged contact between different bacterial species in a protected and stable environment, and continuous exposure to low-dose antibiotics shed by patients, could favour selection and lead to the formation of potentially harmful AMR reservoirs that are very difficult to eliminate [1]. A study of 73 intensive care units (ICUs) in France found that 50.9% of sink drains were contaminated with multidrug-resistant organisms (MDROs) [3].

A recent survey of healthcare workers (HCWs) in Ireland found that awareness of sinks as potential reservoirs of dangerous pathogens was common among infection prevention and control professionals, but not among other professionals [4]. In fact, sinks have been introduced close to patients to improve hand hygiene as part of infection prevention and control measures, and are not perceived as ‘dirty’ places [5]. Many studies in recent years have shown that a waterless (or water-safe) approach in ICUs could reduce nosocomial colonisation and infection rates with gram-negative pathogens [6, 7]. A comparison of infection rates between ICUs with and without sinks in patients’ rooms in Germany showed that patients in ICUs with sinks had a 30% higher risk of nosocomial infections [8].

However, removing sinks from patient rooms or planning ICUs without them can be challenging for HCWs who are used to work with them.

The results presented are part of a larger project funded by the German Federal Government (Bundesamt für Bauwesen und Raumordnung - Project number: 10.08.18.7-20.07) to identify challenges and solutions for future ICU planning. Aim of the multi-centre survey was to investigate some aspects of the ICU built environment (among those, the sinks), which are considered to play a role in infection prevention and control, through the eyes of HCWs. After the survey,  we conducted a small single-centre observational study to verify and better understand the answers given in the multicentre survey about the use of sinks in intensive care units.

## Methods

A non-validated 21-question survey was developed by a study team comprehending one infection control nurse, two infection control practitioners and two architects. The infection control nurse and one of the practitioners had many years of working experience on an ICU. The survey instrument was based on previous observations and personal experience of the study steam about aspects of the ICU built environment which are critical for infection prevention and control (s. Supplement Material).

For the multicentre survey a questionnaire was distributed to seven ICUs in five hospitals (five tertiary care centres, one secondary care centre) in Berlin, Germany, between August and September 2022. In January 2023, another six ICUs in a tertiary care centre in Braunschweig (Germany) participated in the survey. The involved ICUs cover a wide range of specialisations, 6x anaesthesiology, 4x surgical, 3x medical and 2x interdisciplinary. 

The questionnaires were left in an open box on the ward and could be returned in a locked box next to it. All staff could participate. Ethical approval was not required as participation was anonymous and not mandatory.

A medical student and an infection prevention nurses observed sink utilisation in patients’ rooms in three of the ICUs participating in the study (ICU 1, ICU 2, ICU 5) during routine audits for hand hygiene compliance. In these three ICUs 2% Chlorhexidine impregnated clothes for patient body washing and tap water and tooth brushing for oral hygiene are implemented as standard of care.

MS Excel was used to analyse answers. All analyses are descriptive.

## Results

A total of 258 questionnaires were returned. Each participating ICU received an average of 20 questionnaires. One questionnaire was excluded due to incomplete responses.

The size of the ICUs varied from 8 to 30 beds and were managed by different specialties (anaesthesia, surgery, medicine, interdisciplinary). The basic characteristics of the wards participating in the survey and the number of participating subjects per ICU are summarised in Table 1.

Most of the participating HCWs − 223 (87%) - were nurses, 14 (5.4%) were doctors, 7 (2.7%) were physiotherapists and 11 (4.3%) were other professionals.

Overall, 91% of respondents considered it important or very important to have a sink in the patient’s room, but only about 50% reported daily use of the sink for hand hygiene (Table 2).

Out of the 231 HCW considering “very useful” or “useful” to have the sink in the patient room 137 (59%) said to use it everyday for hand hygiene and 49 (20%) to use it never or just occasionally for this purpose. On the other hand out of the 22 who consider the sink not useful only 2 (10%) say to use it everyday for hand hygiene.

In the single-center observational study sink use was observed for 17 h over 8 days. Observations were made for an average of 2 h per day during the morning shift, when most contact is possible due to routine morning tasks such as washing patients’ bodies and teeth. During this time, we recorded 33 individual contacts between the sink and HCW. In 20/33 (60%) of the contacts, dirty water or other waste (gastric tube contents, dialysis fluid, personal hygiene products) was disposed of .

**Table 1:** Main characteristics of participating ICUs

Center	ICU	Number of beds	Specialty	*N*° participants
1 (Tertiary care university hospital)	ICU 1	14 (7 two-bed rooms)	Anesthesiology	27
	ICU 2	10 (5 two-bed rooms)	Surgery	25
2 (Tertiary care university hospital)	ICU 3	21 (9 one-bed rooms, 6 two-bed rooms)	Interdisciplinary	24
	ICU 4	21 (9 one-bed rooms, 6 two-bed rooms)	Interdisciplinary	26
3 (Tertiary care university hospital)	ICU 5	30 (10 one-bed rooms, 2 two-bed rooms, 4 4-bed rooms)	Anesthesiology	30
4 (Secundary care)	ICU 6	18 beds (8 one-bed rooms, 5 two-bed rooms)	Medical	18
	ICU 7	20 Beds (6 one-bed Rooms, 7 two-bed rooms)	Anesthesiology	
5 (Tertiary care, not-university hospital)	ICU 8	13 beds (5 one-bed rooms, 4 two-bed rooms)	Medical	16
	ICU 9	14 beds (6 one-bed rooms, 4 two-bed rooms)	Anesthesiology	
	ICU 10	18 beds (2 one-bed rooms, 8 two-bed rooms)	Anesthesiology	
6 (Tertiary care, not-University hospital)	ICU 11	9 (4 two-bed rooms, 1 one-bed room)	Surgery	91
	ICU 12	10 (4 two-bed rooms, 2 one-bed rooms)	Surgery	
	ICU 13	19 (five 3-bed rooms, one 4-bed room). One 3-bed room for isolation (cohorted)	Surgery	
	ICU 14	8 beds	Anesthesiology	
	ICU 15	17 (7 two-bed rooms, 3 one-bed rooms)	Medical	

in the sink. In a further 7/33 (21%) contacts, the sink was used as a work surface to place materials during care activities. Only 5/33 (15%) contacts used the sink to collect clean water. In 3/33 (1%) it was used for hand washing. Hand disinfection with alcoholic hand rub took place after 7/33 (21%) contacts.

**Fig. 1:**
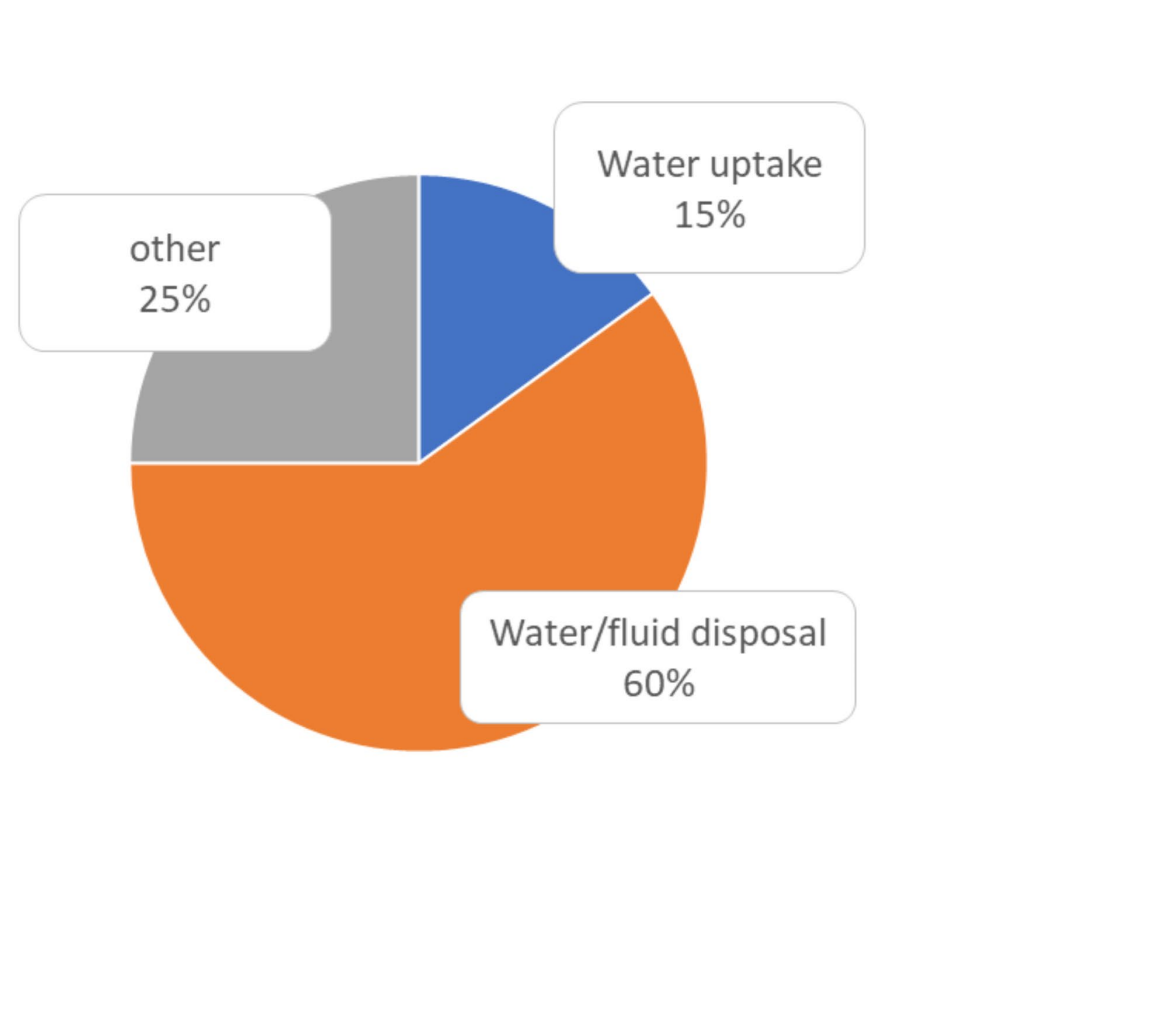
Percentage of Sink contacts for water uptake or water/fluid disposal in the three observed ICUs. Washing hands after patient contact is considered to be disposal of contaminated water

Figure 1; Table 3 summarise sink contacts for clean water uptake, contaminated water/fluid disposal or other activities in the three ICUs observed. Washing hands with water and soap after patient contact, although in line with the WHO 5 moments of hand hygiene, results in the release of pathogens from the HCW’s hands into the sink drain and is therefore considered a disposal of contaminated water.

**Table 2:** Excerpt of the survey questions related to sink usage. *N* = 257

How useful is it in your view to have a sink in the patient room?	%	*N*
Very useful	63,4	161
Useful	27,6	70
Not so useful	6,3	16
Not useful	2,4	6
**How often are you using the sink in the patient room to wash your hands?**	%	
Every day	56	139
Often, but not everyday	17,3	43
Occasionally	18,5	46
Never	8,1	20

**Table 3:** Description of the 33 observed HCW/Sink contacts. HD : hand disinfection

Professional group	Utilization	Removal/disposal	HD before use	HD after use
Nurse	Disposal of beverage residues and rinsing solution from a gastric tube	Water/Fluid disposal	no	Yes
Doctor	Hand washing after sonography/patient contact	Water/Fluid disposal	no	no
Nurse	Water uptake for oral hygiene	Water uptake	no	no
Nurse	Disposal of used bath water	Water/Fluid disposal	no	no
Nurse	Disposal of gastric secretions	Water/Fluid disposal	no	no
Service	Place the cleaning bucket in the sink, dispose of the cleaning water in the sink	Water/Fluid disposal	no	no
Nurse	Placing the patient’s used drinking bottle on the tap	other	no	no
Nurse	Temporarily placing fresh Nurse products on the edge of the sink	other	Yes	no
Nurse	Disposal of used bath water, placing the used bowl in the sink	Water/Fluid disposal	no	no
Nurse	Water uptake for oral hygiene	Water uptake	no	no
Nurse	Rinse wet shaver (used) under running water	Water/Fluid disposal	no	Yes
Nurse	Place a blood collection tube on the edge of the sink after blood collection	other	no	Yes
Nurse	Emptying renal replacement therapy bag in the sink	Water/Fluid disposal	no	no
Doctor	Leaning against the sink during the visit	other	no	no
Nurse	Placing the filled bath bowl in the sink during patient hygiene	other	no	no
Nurse	Placing used cup of the patient	other	no	no
Nurse	Emptying used bath water into the sink	Water/Fluid disposal	no	no
Nurse	Water uptake for oral hygiene	Water uptake	no	no
Nurse	Washing medication grinder after use	other	no	no
Nurse	Emptying renal replacement therapy bag in the sink	Water/Fluid disposal	no	no
Nurse	Washing medication grinder after use	other	Yes	Yes
Nurse	Hand washing after patient contact	Water/Fluid disposal	Yes	Yes
Nurse	Disposal of bath water	Water/Fluid disposal	no	no
Nurse	Knocking out the patient’s comb (used).	Water/Fluid disposal	no	no
Nurse	Hygienic hand washing after patient contact	Water/Fluid disposal	no	Yes
Nurse	Disposal of bath water after hair and body washing. Rinsing out the used wash bowl	Water/Fluid disposal	no	no
Nurse	Water uptake of bath water	Water uptake	no	no
Nurse	Hygienic hand washing after patient contact	Water/Fluid disposal	no	Yes
Nurse	Water uptake for oral hygiene	Water uptake	no	no
Nurse	Disposal mouth rinsing solution	Water/Fluid disposal	no	no
Nurse	Rinse wet shaver (used) under running water	Water/Fluid disposal	no	no
Nurse	Cleaning material under running water	Water/Fluid disposal	no	no
Nurse	Disposal of bath water	Water/Fluid disposal	no	no

## Discussion

Healthcare water environments can be reservoirs for hospital-acquired pathogens because the water environment favors the formation of biofilm, which is largely unaffected by normal decontamination efforts. As a result, sinks and drains can be an ongoing source of pathogens´ transmission [9], but HCWs and health facility planners don’t seem to be fully aware of this [4].

Sinks in patient rooms have been suggested a way to provide an easily accessible option for hand hygiene [10]. Meanwhile alcohol-based hand rubs are the preferred method for hand hygiene due to their higher antimicrobial efficacy [11] with the exceptions that hands should be washed with soap and water when they are visibly soiled or when exposure to potential spore-forming pathogens is strongly suspected or proven, including during outbreaks of *C. difficile*.

Sinks are used for many different activities other than hand hygiene (such as collecting water for daily patient care and disposing of human waste). Grabowsky showed that 17% of contacts between HCW and the sink in an north American ICU were for hand hygiene (4% specifically for hand washing). Rather, various non-hand hygiene specific activities were regularly carried out, from medical care measures (syringe preparation, emptying intravenous solutions, etc.) to processing patients’ own food or drinks and cleaning (cleaning the sink etc.) [12]. Many of these activities imply the discharge of human waste into the drain, which was identified as a major risk factor for sink contamination [3].

Avoiding tap water for patient care could therefore be a first step to reduce the need of sinks in patient proximity and different strategies of water-less care have been successfully implemented to stop sink-related outbreaks [13]. Even the isolated use of antiseptic wipes instead of water and soap to wash patients has been effective in reducing central line associated blood stream infections (CLABSI) [14, 15] and acquisition of multiresistant bacteria [16]. Nevertheless, waterless care struggles to be accepted by HCW. There are widespread concerns about acceptability to patients for cleansing wipes and dry shampoo, the possible spread of gastroenteric pathogens (such as CDI) and logistic and sustainability challenges (delivery, storage, increase of waste) [17].

In Germany, the German Commission for Hospital Hygiene (KRINKO) and the German Interdisciplinary Council for Intensive Care (DIVI) recommend that new hospital buildings should not include washbasins in patient rooms in high-risk areas, such as intensive care units [18, 19]. However, according to a survey, 80% of existing ICUs still have sinks in patient rooms and 84% use tap water for patient care [20].

Existing practices and habits are a major challenge to implementing infection prevention measures. Over 90% of respondents to our survey said that having a washbasin in the patient’s room was ‘useful’ to ‘very useful’. This finding is very interesting because it highlights the difficulty of translating scientific evidence into everyday practice. The fact that up to 20% of respondents who consider sinks useful, declare not to use it for hand hygiene on a daily basis, confirms that also in our cohort sinks are used for different purposes.

In our single-center observations, we found that the majority of sink contacts involved the disposal of potentially contaminated materials. It therefore makes sense not to use the same sinks for ‘clean’ activities, such as taking water for patient hygiene. Extra Hand washing stations, out of the patient area, should be reserved only for HCWs use. Other considerations, such as the design of handwashing sinks and drains, should be considered when planning water systems in healthcare facilities to minimize the risk of environmental contamination and therefore risk to patients [21, 22].

This study has limitations. Firstly, the total population is not known, as we are not aware of the total number of HCW working on each ICU during the study period. Therefore, no answer rate can be calculated. Secondly, We were able to observe only for a mean time of two hours a day and we recorded a mean of about 2 HCW/Sink contacts per hour. Observations on a longer time period should be conducted to better characterize frequency and typology of sink utilization. In the multicentric survey, the reason why HCW consider sinks very useful other than for handwashing were not further examined and no information on standard of care (body washing, oral care, usw.) were reported. This information could give further understanding about the needs of HCW and how to meet these needs without sinks. The observational study was carried on in a limited number of ICUs in a single center, although this strongly limits generalizability, our findings don´t differ much from those of previous studies [12] and can give – we think – an useful insight on the different activities happening around sinks in an ICU. Despite a water-less strategy for patient washing in the three observed ICUs, the use of water and soap for patient body care was still common. The non-compliance to impregnated clothes can be explained by a low awareness of risk associated with tap water among non-IPC personnel, as underlined by Kearney et al. [4].

Although there is an international trend to avoid sinks in intensive care units, it will be a long time before this change is complete. As long as sinks are considered necessary in routine care, different strategies of minimizing risks can be implemented: (1) sinks should be designed as “one-way” systems, i.e. separate sinks for water uptake and water discharge, (2) alternative handwashing stations out of the patient area exclusively for HCW should be provided, (3) the use of an alcoholic hand rub after each contact with the sink should be encouraged to prevent further transmission, (4) education on not to use the sink as a surface to storage clean equipment should be continuously provided, with audits and feed-backs to enhance compliance, (5) training and education to increase acceptance of water-less or water-safe care are important were this standard is implemented, (6) development of national guidance on waste water systems can impact understanding of water care from clinical and planning teams, toward a risk-based approach.

## Data Availability

No datasets were generated or analysed during the current study.
